# 0735. Effects of metoprolol in a porcine model of septic shock

**DOI:** 10.1186/2197-425X-2-S1-P57

**Published:** 2014-09-26

**Authors:** AL Corrêa, DT Fantoni, JOC Auler, NG Queiroz-Hazarbassanov, CO Massoco, DA Otsuki

**Affiliations:** Department of Surgery, Faculdade de Medicina da Universidade de São Paulo, São Paulo, Brazil; Department of Surgery, Faculdade de Medicina Veterinária e Zootecnia da Universidade de São Paulo, São Paulo, Brazil; Department of Pathology, Faculdade de Medicina Veterinária e Zootecnia da Universidade de São Paulo, São Paulo, Brazil

## Introduction

The use of cardiovascular drugs increased in recent years [1], including the use of β-blockers, such as metoprolol. And although the β-blockade is still very contradictory in patients with sepsis, both studies in experimental models and in septic patients with previous prescription of β-blockers have shown a potential role of these drugs in patients with sepsis [2, 3].

## Objectives

To evaluate the effect of metoprolol, a beta 1 selective beta-blocker, over echocardiographic parameters, cytokines and cardiac markers, in a porcine model of septic shock.

## Methods

Twenty pigs in which septic shock was induced through intravenous *E. coli* infusion (6x10^9^ c.f.u/kg in 2h) were randomly assigned (n=10 per group) to the Metoprolol group (214.2 µg kg^-1^ of metoprol infused in 45 minutes) or Control group, which received a correspondent volume of normal saline. Transesophageal echocardiographic (TEE) parameters were recorded at baseline, T120 (end of bacteria infusion), T180, T240, T300 and T360 minutes. Blood samples obtained at baseline, T120 and T360, were analyzed for cytokines (tumor-necrosis factor (TNF)-alpha, interleukin (IL)-1beta, IL-6 and IL-10), troponin I (TNNI) and B-type natriuretic peptide (BNP) using commercial ELISA kits. A resuscitation protocol with lactated Ringer's solution, norepinephrine and dobutamine was used during the study to maintain the mean arterial pressure > 65 mmHg, the central venous pressure between 8-12 mmHg and the mixed venous oxygen saturation > 65%. TEE and IL-10 results were compared using the two-way repeated measures ANOVA. The nonparametric data were analyzed with the Kruskall-Wallis and the Mann-Whitney U test.

## Results

The TEE parameters showed a reduction of the left ventricular diastolic and end-systolic volumes (p< 0.001) and areas (p< 0.001) in both groups after sepsis induction. No alteration was observed on the measures of left ventricular ejection fraction. Both groups presented a similar behaviour of all cytokines, with a significant elevation after bacteria infusion (p< 0.001). Although both groups have shown an increase in TNNI, only in the Control group this increase was significant (p=0.002). No difference in the BNP values was observed between timepoints or between animals receiving metoprolol or normal saline.

## Conclusions

The administration of metoprolol did not improve echocardiographic parameters, cytokines or cardiac markers in pigs with septic shock. And despite the lack of beneficial effects of this drug, in a scenario where the administration of β-blockers is increasing constantly, it is important to know that its administration is well tolerated in these patients.
